# Predictors of protein losing enteropathy after Fontan completion: An 8-year retrospective study at Sheikh Khalifa Medical City

**DOI:** 10.21542/gcsp.2023.17

**Published:** 2023-08-01

**Authors:** Antoine Fakhry AbdelMassih, Laszlo Kiraly, Hazem El Badaoui, Mohammad Khan, Balazs Hetharsi, Judit Noemi Till, Aleksandr Y. Omelchenko, Alaa Ziad Salah, Farah Tarik Al Jburi, Laila Alkhouli, Mina Taher, Najah Alhosani, Omnia Youssef, Sumaiya Iqbal, Zahraa Allami, Neerod Kumar Jha, Eman Mahmoud Hamad, Yasmin Omar, Arshad Khan, Zafar Azeez, Michael Attia, Mariam Mina, Alyaa Al Ali, Yara Khaled Afifi, Meryam El Shershaby, Afnan Musleh

**Affiliations:** 1 Pediatric Cardiology unit, Department of Pediatrics, Faculty of Medicine, Cairo University, Cairo, Egypt; 2 Pediatric Cardiology Division, Cardiac Sciences’ department, Sheikh Khalifa Medical City, Abu Dhabi, UAE; 3 Pediatric Cardiac Surgery at Department of Cardiac, Thoracic and Vascular Surgery, National University Hospital; 4 Pediatric Cardiology Department, Sheikh Shakhbout Medical City, Abu Dhabi, UAE; 5 Pediatric Cardiac Intensive Care Department, Sheikh Khalifa Medical City, Abu Dhabi, UAE; 6 Pediatric Cardiac surgery Division, Cardiac Sciences’ department, Sheikh Khalifa Medical City, Abu Dhabi, UAE; 7 Pediatrics’ Department, Sheikh Khalifa Medical City, Abu Dhabi, UAE; 8 Pediatric Echocardiography, Sheikh Khalifa Medical City, Abu Dhabi, UAE; 9 Cardiology department, Sheikh Khalifa Medical City, Abu Dhabi, UAE; 10 Pediatrics’ department, Danat Al Emarat Hospital for Women & Children, Abu Dhabi, UAE; 11 Yas Clinic Group, Abu Dhabi, UAE

## Abstract

Background: The Fontan procedure is the final stage of a three-stage palliation process in patients born with a univentricular heart as part of hypoplastic left heart syndrome (HLHS) or other pathologies with a univentricular heart. As essential as this procedure has proven to be for such cases, the Fontan physiology diminishes cardiac output and expands systemic venous pressure, which then leads to venous congestion that can be complicated by protein-losing enteropathy (PLE). This retrospective study aimed to identify the predictors of such complications in all patients who underwent completion of the Fontan procedure at our center (Sheikh Khalifa Medical City/SKMC) in the past eight years.

Methods: This study examined the medical records of patients who underwent completion of Fontan repair at our center since the inauguration of the cardiac surgery program of SKMC in the United Arab Emirates (UAE) – 01 Jan 2012 to 31 Dec 2020. Exclusion criteria included the absence of any of the undermentioned data in patient files. Patients were divided into two groups: those who developed PLE and those who did not. For each group, the following data were collected: demographics data (current age and age at completion of Fontan), clinical and laboratory data (oxygen saturation, serum albumin), echocardiographic data (classification of original cardiac diagnosis, degree of atrio-ventricular valve regurgitation, ventricular functions), hemodynamic data (mean pressures of superior vena cava and pulmonary arteries before Fontan completion), operative data (type of initial palliation, type of Fontan, presence of fenestrations and its size) and the need for any cardiac intervention prior to Fontan completion, such as atrio-ventricular valve repair, peripheral pulmonary stenting and arch balloon dilatation.

Results: Of the 48 included patients,13 (25%) developed PLE. Multivariate regression analysis proved that the best predictors of PLE were superior vena cava mean pressure (*P* = 0.012) and the degree of atrio-ventricular valve regurgitation (*P* = 0.013). An oxygen saturation <83% prior to Fontan completion was 92% sensitive in predicting PLE after Fontan completion.

Conclusion: This is a single-center study of the predictors of PLE after Fontan procedure and, as expected from similar studies, SVC pressure higher than 11 mmHg and moderate-to-severe atrio-ventricular valve regurgitation were predictors of Fontan failure. The higher prevalence of PLE in our cohort, as well as lower cut-offs of SVC pressure that can predict complications, may be related to the predominance of hypoplastic left heart in the operated patients, which has been the main referral center for cardiac surgeries in UAE in the last decade.

## Background

Francis Fontan designed the Fontan Circulation in 1971 to overcome the absence of two separate cardiac ventricles. This procedure was originally aimed at syndromes involving a single left ventricle. However, after recent modifications, it is also applicable for cases with a single right ventricle, such as those associated with hypoplastic left heart syndrome(HLHS)^1^.

Nevertheless, owing to the resulting systemic venous congestion and low cardiac output, complications following the Fontan procedure are not uncommon^2^. The most important and disabling complication due to systemic congestion is the intestinal lymphangiectasia and subsequent loss of protein-rich lymph, leading to hypoalbuminemia and repeated hospital admissions due to generalized edema and dependency on long-term albumin infusion^2,3^.

Morbidity following Fontan is now only ≤5%, compared with 15–30% in earlier decades, with survival at 20 years being 85%^4^. The overall incidence of PLE following Fontan ranges between 12-15%, however a higher incidence of PLE (17-20%) was observed in patients with systemic right ventricle, namely, HLHS^5^.

A systemic right ventricle has a higher likelihood of developing moderate-to-severe atrioventricular (AV) regurgitation and ventricular dysfunction, with subsequent pulmonary venous congestion, resulting in increased pulmonary vascular resistance and decreased flow in cavopulmonary shunts. The latter mechanism might explain the higher incidence of PLE encountered in HLHS with the completed Fontan pathway.

The first pediatric cardiac surgery program in the UAE was inaugurated in SKMC a decade ago, the goal of which was to provide residents of the country with the needed cardiac surgical and interventional options and to gradually abolish the need for them to travel abroad and the ensuing economic burden resulting from this need. For at least eight years since its creation, it remained the only referral center in the UAE until two other centers were developed in Dubai and Sharjah.

The primary outcome parameter of our retrospective study was to determine the prevalence of Fontan failure in the form of protein-losing enteropathy (PLE) in our center for cardiac surgery, and the secondary outcome parameter was to determine the best predictor of such failure among the different risk factors. These include the length to follow-up, sex, type of original cardiac lesion, pulmonary vascular resistance, dominant ventricle, extent of AV valve regurgitation, and pressure cut-offs in the SVC that can predict Fontan failure.

## Methods

### Study subjects

Medical reports of all patients who underwent Fontan in the period between 01 Jan 2012 and 31 Dec 2020 in our center were retrieved from the CERNER enterprise system (Millennium: Cerner Corporation, Kansas City, Missouri).

Exclusion criteria included: (1) all patients who had reverted their Fontan pathway to a biventricular repair or a one-and-a-half repair due to advances in the available surgical techniques, and (2) patients with incomplete medical records whose files did not include the undermentioned collected variables.

### Study Methods

The following data were retrieved from the system.

Demographic and clinical data:

• *Presence or absence of PLE*: PLE was defined by the presence of hypoalbuminemia (<30 g/L)^6^ and clinical symptoms of documented hypoproteinemia (e.g. edema, effusions), after exclusion of other causes as hepatic and renal causes^6^.
• *Age at development of PLE*
• *Age at completion of Fontan and current age with subsequent deduction of duration from Fontan completion*
• *Sex*
• *Development of PLE*

Operative data:

• *Type of initial palliation: such as Norwood-Sano in hypoplastic left heart syndrome, Blalock-Taussig shunt in pulmonary atresia, and sole pulmonary artery band in cases with double-inlet left ventricle.*
• *Type of Fontan (all patients performed an extracardiac conduit)*
• *Conduit size*
• *Presence of fenestration*
• *Size of fenestration*
• *The need for any pre-Fontan cardiac interventions such as peripheral pulmonary artery stenting (seen in cases with underlying pulmonary atresia), arch balloon dilatation (needed in some instances post-Norwood) and AV valve repair.*
• *Type of anticoagulants/antiplatelets used*

Hemodynamic data: (by invasive hemodynamics in the most recent cardiac catheterization before completion of Fontan)

• *SVC (Glenn) pressure*
• *Peripheral PA mean pressure (calculated as the average pressure of the mean left and right pulmonary artery pressures)*

Echocardiographic data:

• *Degree of AV valve regurgitation*

Assessment of valvular regurgitation using jet area^7^:

• <20% of the atrium: Mild regurgitation
• 20–39% of the atrium: Moderate regurgitation 
• >40% of the atrium: Severe regurgitation

Degree of main ventricle dysfunction:

Ventricular dysfunction was assessed qualitatively by two independent operators^7^.

• Classification of the original cardiac diagnosis based on: Pulmonary overflow vs. lesions with decreased pulmonary blood flow.
• Dominant right (as in hypoplastic heart syndrome) vs. dominant left ventricle (as in tricuspid atresia, pulmonary atresia).

### Statistical analysis

Data were analyzed using MedCalc and Excel software. Patients were divided into two groups: Group 1: Fontan patients who developed PLE, and Group 2: Fontan patients who did not develop PLE.

Normally distributed numerical variables were presented as Mean and Standard deviations. Inter-group differences were assessed using the unpaired *t-*test. Categorical data were compared using the Fisher’s exact test rather than the Chi-squared test as some of the groups’ frequencies were < 5%.

Multivariate analysis was performed to determine the best predictor of PLE in the Fontan patients. Receiver Operating Characteristic (ROC) was performed to determine the sensitivity and specificity of the best predictors, shown by multivariate analysis in predicting PLE after Fontan. Interactive dot diagrams and classic ROC curves were designed to illustrate results of the ROC analysis.

## Results

Our study included 48 patients who underwent Fontan completion. The mean age at completion of Fontan was 3.9 ± 1.4. Fontan technique was uniform across all patients, performed as a fenestrated extracardiac conduit, with a conduit size varying between 16 and 20 (predominantly 18). Warfarin was given as an anticoagulant for six months postoperatively, thereafter switching to aspirin.

**Table 1:** Overall demographic and surgical data of the study cases.

Variables		
Total number of Cases: 48		
Age (mean ± SD)		8.9 ± 5
Presence of PLE N (%)		13 (27)
Age at completion of Fontan (mean ± SD)		3.9 ± 1.4
Gender	Female N(%)	16 (33)
	Male N(%)	32 (67)
Length to Follow up (mean ± SD)		4.9 ± 2
Underlying Cardiac Diagnoses N (%)	Hypoplastic left heart Atresia	16 (33)
	Pulmonary atresia variants	23 (48)
	Others (Double inlet left ventricle, unbalanced atrioventricular canal)	9 (19)
Type of initial palliation N (%)	Norwood-Sano variant (HLHS)	16 (33)
	Norwood-BT shunt variant (HLHS)	0 (0)
	BT shunt (Pulmonary atresia variants)	23 (48)
	PAB (DILV/Unbalanced AVC and others)	9 (19)
Type of Fontan N (%)	Extracardiac conduit	48 (100)
	Lateral Tunnel	0 (0)
	Atrio-pulmonary connection	0 (0)
Size of Fontan Conduit (mean ± SD)		18.2 ± 0.9
Presence of Fenestrations		48 (100)

The initial palliative stage was Norwood-Sano in 16 patients (with hypoplastic left heart syndrome), the Norwood-BT shunt was not used, a Blalock-Taussig shunt (in pulmonary atresia variants) was implanted in 23 patients and a pulmonary artery band in 9 patients (such as double inlet left ventricle, unbalanced atrio-ventricular canal defects and others).

There was a relatively high prevalence of PLE (27%), in the studied cases Table 1.

We compared of the demographic, operative, echocardiographic and hemodynamic data between the two study groups (Group 1 included the thirteen Fontan patients who developed PLE and Group 2: the 35 patients who did not develop PLE). Details are shown in Table 2.

The age was comparable between both groups, 7.9 ± 2 for Group 1 and 8 ± 4 for Group 2. The length to follow-up is seemingly unrelated to the development of PLE, as it was similar between the two groups. Neither the size of the Fontan conduit nor the size of fenestrations impacted its outcome, with P values of 0.4 and 0.39 respectively between the two study groups.

**Table 2:** Comparison of the epidemiologic, echocardiographic, operative and hemodynamic parameters between the two study groups.

Variables			Group 1 (*N* = 13)	Group 2 (*N* = 35)	*P* Value
Demographic	Age (mean ± SD)		7.9231 ± 2.9000	8.6857 ± 4.0784	0.2
	Sex N (/%)	Female	5(38)	11 (31)	0.4
		Male	8 (62)	24 (69)	
	Age at completion of Fontan (mean ± SD)		3.9 ± 2.0108	3.9371 ± 2.1365	0.9
	Length to follow up. (mean ± SD)		3.9308 ± 2.6088	4.7486 ± 3.6733	0.2
Clinico-laboratory data	Serum Albumin (g/L)		21 ± 2	37 ± 2	**<0.001** ^*^
	Oxygen saturation before completion of Fontan (mean ± SD)		80,5 ± 5	85 ± 5	**0.004** ^*^
Invasive hemodynamic data prior to Fontan	SVC mean pressure (mean ± SD)		13.7 ± 1.4	9.7 ± 3.4	**0.002** ^*^
	PA mean pressure (mean ± SD)		11.5 ± 3.4	9 ± 3.7	**0.04** ^*^
Operative and peri-operative data	Type of initial palliation And underlying cardiac diagnoses) N (/%)	Norwood-Sano variant (HLHS)	7 (54)	9 (26)	**0.01**
		Norwood-BT shunt variant (HLHS)	0 (0)	0 (0)	
		BT shunt (Pulmonary atresia variants)	2 (15)	21 (60)	
		PAB (DILV/Unbalanced AVC and others)	4 (31)	5 (14)	
	Type of Fontan N (/%)	Extracardiac conduit	13 (100)	35 (100)	**1**
		Lateral Tunnel	0 (0)	0 (0)	
		Atrio-pulmonary connection	0 (0)	0 (0)	
	Presence of fenestration N (/%)		13 (100%)	35 (100%)	**1**
	Size of Fontan Conduit (mean ± SD)		18.7 ± 1	18 ± 0.9	0.4
	Size Fontan Fenestration (mean ± SD)		2.8 ± 1.7	3.4 ± 1.4	0.39
	Need for cardiac interventions pre-Fontan. N (/%)	AV valve repair	1 (8)	0 (0)	**0.2**
		PA stenting	1 (8)	2 (6)	**1**
		Arch balloon dilation	3 (23)	0 (0)	**0.001**
Echocardiographic data	Underlying cardiac lesion According to the presence or absence of pulmonary overflow N (/%)	Pulmonary Overflow lesions	11 (85)	15 (43)	**0.02**
		Decreased Pulmonary blood flow lesions	2 (15)	20 (57)	
	Underlying cardiac lesion According to the dominant ventricle N (/%)	Right ventricle	5 (38)	26 (74)	**0.03**
		Left ventricle	8 (62)	9 (26)	
	Degree of AV regurge N (/%)	None or mild AV valve regurge	4 (31)	28 (80)	**0.001**
		Moderate AV valve regurge	7 (54)	7 (20)	
		Severe AV valve regurge	2 (15)	0 (0)	
	Degree of ventricular dysfunction N (/%)	Normal functions and mild ventricular dysfunction	6 (46)	32 (91)	**0.001**
		Moderate to severe Ventricular dysfunction	6 (46)	3 (9)	
		Severe Ventricular dysfunction	1 (8)	0 (0)	

**Notes.**

Abbreviations AV Atrioventricular N Number PA Pulmonary Artery SD standard deviation SVC Superior vena cava

* Significant P values.

Since all Fontan procedures were done using the same technique as mentioned earlier, there was no statistically significant difference in the operative data between the two study groups, apart from the initial palliative surgery; the initial palliative stage in Group 1 was mostly Norwood-Sano accounting for 54% of its patients, while Blalock-Taussig (BT) shunt was the predominant surgery (60%) seen in patients without PLE. The latter findings are largely not related to the initial surgical palliation, but rather to the underlying anatomy, where patients with hypoplastic left heart syndrome (whose palliation is via Norwood-Sano) were more likely to develop PLE.

Moderate and severe AV valve regurgitations were predominant in patients who developed PLE (69%), while no or mild AV valve regurgitation was most seen in Group 2, accounting for 80% of the group. No patients in Group 2 displayed severe AV valve regurgitation. Similarly, moderate to severe ventricular dysfunction was most seen in patients with PLE (Group 1), accounting for 54% of the total group members, while most of the patients in G2 had normal ventricular functions.

PA mean pressure was higher in Group 1 compared to Group 2 (11±3 vs. 9±3), and SVC pressure difference was even more marked between Group 1 than Group 2 (13±1 vs. 9±3).

Multivariate analysis of the variables with statistical significance by *t*-test and Fisher test between the study groups was performed to determine which was the best predictor of PLE (Table 3). This exercise revealed that SVC pressure had the highest statistical significance, followed by the degree of AV valve regurgitation. Despite the statistically significant difference noted between the two study groups for oxygen saturation, PA pressures, and dominance of RV vs. LV, none of them proved to be a strong predictor of failing Fontan.

**Table 3:** Multivariate analysis for the best predictors of PLE in the study subjects:

Independent variables	Coefficient	Std. Error	*t*	*P*
	-0.3505			
Degree of AV valve regurge	0.2322	0.08957	2.593	**0.0135**
Dominant RV	0.2536	0.1862	1.362	0.1811
Oxygen saturation prior to Fontan	-0.001689	0.008326	-0.203	0.8403
PA mean pressure	-0.01419	0.02096	-0.677	0.5024
Pulmonary overflow in the pre-existing congenital cardiac anomaly	-0.3152	0.3126	-1.008	0.3197
SVC mean pressure	0.05856	0.02218	2.640	**0.0120**
Type of initial palliation	-0.2179	0.1561	-1.395	0.1710
Ventricular Functions	0.2307	0.1372	1.681	0.1010

**Notes.**

Abbreviations AV Atrioventricular PA Pulmonary Artery PLE protein losing enteropathy RV right ventricle SVC Superior vena cava

An interactive dot diagram was constructed to determine the cut-off SVC pressure that could predict the development of PLE. SVC pressure of >11 mmHg was 100% predictive of a negative outcome (Figure 1A) and the sensitivity of SVC pressure to predict PLE was illustrated by the classic ROC curve (Figure 1B).

**Figure 1A.**
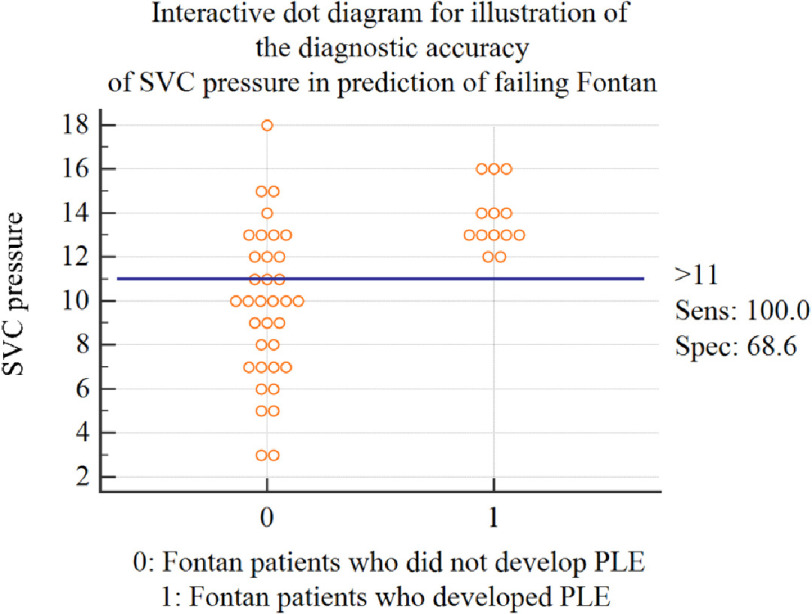
Interactive dot diagram for illustration of the diagnostic accuracy of SVC pressure in the prediction of Fontan failure. *Abbreviations: PLE, protein losing enteropathy; SVC, Superior Vena Cava; Sens, sensitivity; Spec, Specificity.

**Figure 1B.**
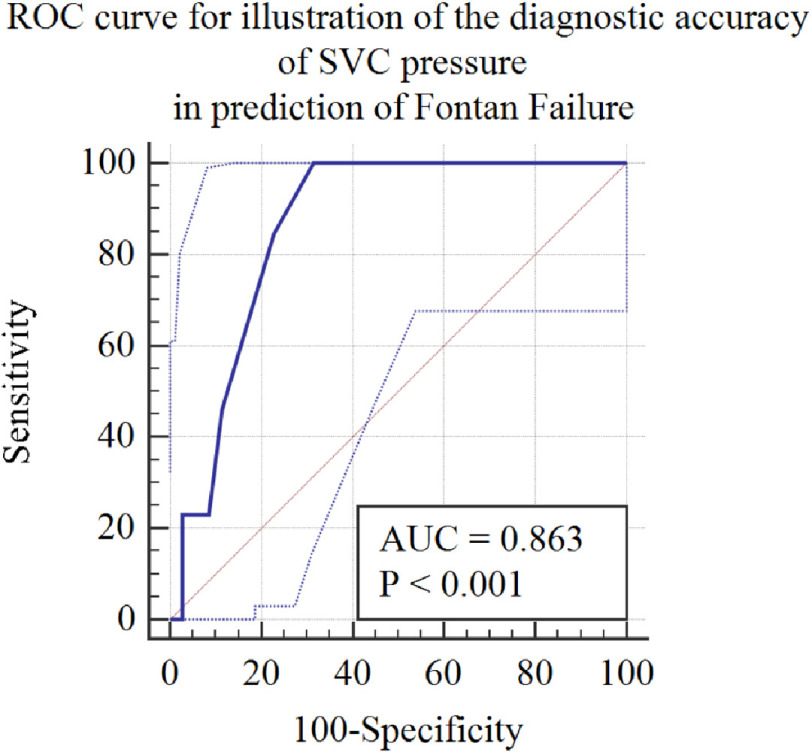
ROC curve for illustration of the diagnostic accuracy of SVC pressure in prediction of Fontan failure. *Abbreviations: ROC, Receiver Operator Characteristic; SVC, Superior Vena Cava; AUC, Area Under ROC Curve.

**Figure 2.**
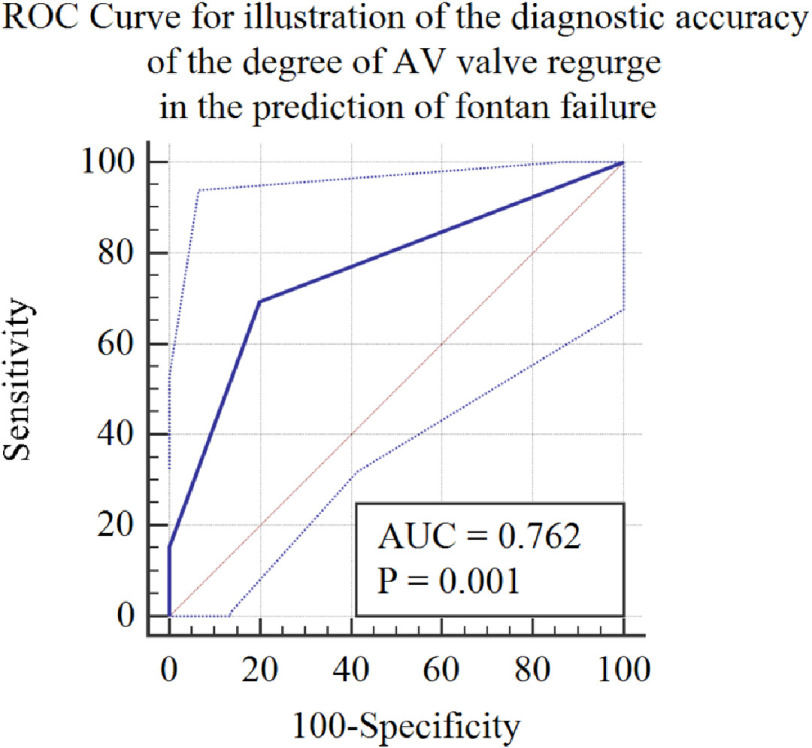
Receiver operating curve (ROC) for illustration of the diagnostic accuracy of AV valve regurgitation in the prediction of Fontan failure. *Abbreviations: AUC, area under the curve; AV, Atrioventricular.

A ROC curve was designed to illustrate the sensitivity of the second-best predictor, namely the degree of AV valve regurgitation, in predicting Fontan failure. Despite having a lower sensitivity of 69%, it achieved a greater specificity in predicting PLE after Fontan procedure (80%) than SVC pressure (Figure 2).

**Figure 3.**
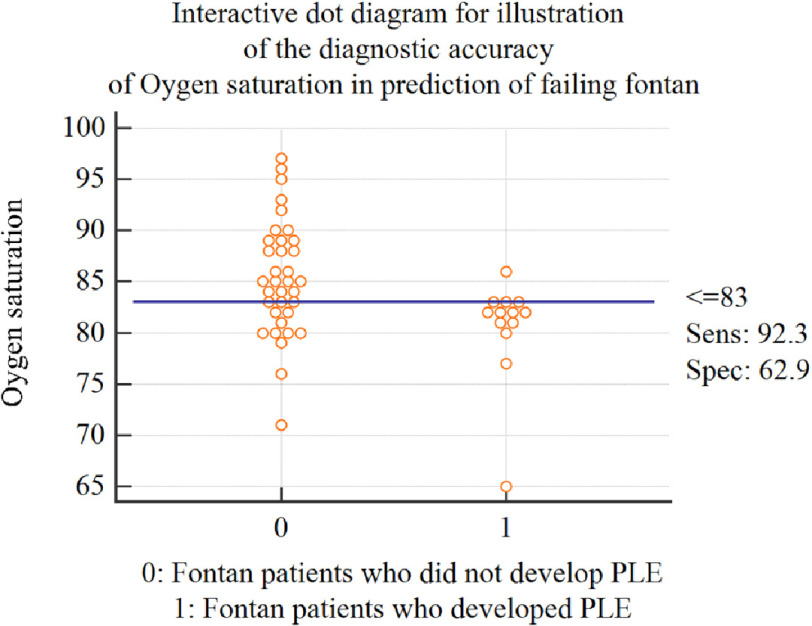
Interactive dot diagram for illustration of the diagnostic accuracy of oxygen saturation in the prediction of Fontan failure. *Abbreviations: PLE, protein losing enteropathy; SVC, Superior Vena Cava; Sens, sensitivity; Spec, Specificity.

Finally, yet importantly, oxygen saturation, which is an easy bedside predictor, achieved a high sensitivity of 92% in predicting PLE after Fontan procedure, despite not being one of the best predictors of PLE in multivariate regression (Figure 3).

## Discussion

Our study pointed to a higher prevalence of PLE in our cohort of Fontan patients (27%) compared to a prevalence of 15% in most other reports^8^. The predominant diagnosis in patients diagnosed with PLE was HLHS, and the subsequent structural associations with this complication was an incompetent AV valve.

The study of predictors of PLE after Fontan is not a new topic in the literature, however it is important, to revise the “dos and don’ts” and the hurdles faced in optimal surgical outcomes.

PLE is one of the most serious postoperative complications and is consequently a powerful predictor of morbidity and mortality in patients after the Fontan operation. It is important to recognize the risk factors that might predict the occurrence of this complication, in order to be able to eliminate them when possible, or to avoid the Fontan procedure in patients with any of those red flags^9^.

Our research might be the second single-center experience in the Gulf region concerning patients who underwent Fontan completion following an epidemiologic study by Najash et al. in 2021 that depicted the rate of short- and long-term complications from a univentricular pathway. The latter only discussed the rate of complications, rather than the respective predisposing factors^10^. Despite the development of multiple esteemed pediatric cardiac surgery hubs in the region, the scarcity of published reports reflects the need to improve documentation and publications in the region.

As mentioned earlier, one of the most striking findings in our study is the higher rates of PLE in our patients compared to similar studies, which reported a 27% incidence of PLE, while most other reports showed a lower incidence, ranging between 12-15%. A recent report by Bernardi et al.^5^ showed a similar incidence of PLE as in our study; this specific incidence was encountered in patients with a predominant right ventricle.

The reason for this finding might be related to the nature of the referral center. For years, as mentioned earlier, it has remained the only center to perform staged univentricular repair for HLHS. HLHS is invariably associated with pulmonary overflow and moderate-to-severe AV valve regurgitation, which are the two important predisposing factors for increased venous pressure and failing Fontan^5^.

There is no fixed time between Fontan surgery and the development of PLE, however 30-50% patients tend to develop this complication after five years of the procedure. In our study, 27% of the patients developed PLE following Fontan, with an average duration of 2.4 years between Fontan and the initial manifestations of PLE, which is shorter than the numbers previously reported. The length to follow-up was comparable between the two study groups, which signifies that the length to follow up is not a key factor in the development of PLE and that PLE might develop earlier if the risk factors needed for its development exist^11^.

Arterial oxygen saturation is a simple noninvasive measure that has shown high sensitivity and specificity in our study as a predictor of post-Fontan PLE. Intriguingly, our study is not the first to demonstrate the importance of oxygen saturation as a predictor of short- and long-term complications after Fontan surgery. Loomba and colleagues^12^ demonstrated that Fontan completion should be done before the arterial oxygen saturations decrease to 82%. Below 82% there was a high likelihood of increased length of hospitalization. It is still unclear how lower oxygen saturation can cause increased venous congestion; a suggested mechanism is through a direct increase in pulmonary vascular resistance via induction of pulmonary vasoconstriction^13^. Low oxygen saturation prior to Fontan might also result from Fontan dysfunction, leading to increased flow across the fenestration with more desaturation^14^.

Fontan circulation is a low-energy circulation, even during exercise. Larger conduits were proven to have redundant spaces with subsequent development of venous congestion due to loss of energy. Our study could not reveal a significant difference in conduit size between the two study groups, as most of the conduits used were between 16–18 mm. However, patients developing PLE had a statistically insignificant conduit size (18.7 mm which might confirm the hypothesis of loss of energy through larger conduits^15^.

Pre-Fontan assessment of SVC and pulmonary pressure is essential to determine whether hemodynamics are suitable for Fontan. Pulmonary overflow and an incompetent tricuspid valve lead to severe regurgitation and consequent pulmonary venous congestion, which, in turn, increases pulmonary vascular resistance. The latter mechanisms are invariably associated with Fontan failure as they impair venous return against elevated PA pressures. Consequently, HLHS is the most prevalent lesion associated with PLE following Fontan in our studies as well as in several others. Cut-offs for SVC pressures higher than 13 mmHg denoted a high risk for the completion of Fontan. In this study, an SVC pressure > 11 mmHg was 100% sensitive and 68% specific in predicting the occurrence of PLE post-Fontan^16^.

The reason for the lower SVC cut-off in our cohort of patients may also be related to the predominance of HLHS patients compared to other diagnoses. Intriguingly, it has been found that pulmonary overflow leads to increased pulmonary vascular resistance, which will be subsequently reflected in SVC pressure, but also a higher pulmonary blood flow and the association of bilateral SVCs with HLHS might lead to a relatively smaller caliber of right SVC due to distribution of venous return from the head and neck through two veins rather than one^17^. The same cohort of patients have unobstructed pulmonary blood flow leading to a relatively large caliber of peripheral pulmonary arteries. The latter finding might lead to anastomotic gradients between the SVC and pulmonary arteries, thereby increasing SVC pressure. This finding might also explain why the SVC pressure in Group 1 was higher than the mean pulmonary pressure^18^.

In addition, SVC pressure measurement depends on the site of the catheter tip (proximal or distal to the SVC-PA anastomosis). If it is distal to the anastomosis, it will reflect the PA pressure; however, if it is proximal, it will be affected by the gradient across the SVC-PA anastomosis. Therefore, SVC measurements should be interpreted cautiously in the presence of any degree of obstruction across the Glenn anastomosis. In our cohort of patients, there was no significant anastomotic gradient (> 2 mmHg) between the SVC and PA.

Severe AV valve regurgitation was the second-best predictors of PLE following Fontan, which is usually encountered in HLHS where the dominant ventricle is the right ventricle. In HLHS, a small septal leaflet and anterior leaflet prolapse are associated with the development of tricuspid valve regurgitation^19^. Tethering of the tricuspid valve can also occur functionally because of lateral displacement of the anterior PM due to the abnormal geometry and dilation of a single right ventricle in HLHS. In our study only one patient had a trial of repair of the severely regurgitant tricuspid valve, which could not reduce the extent of regurgitation^20^.

Two other interventions needed in patients completing univentricular repair are balloon angioplasty of the arch and balloon dilation/stenting of the pulmonary arteries. In our cohort of patients, three patients in Group 1 (with PLE) required arch balloon dilation, while two patients in Group 2 required stenting of the PAs (specifically the LPA). Residual arch abnormalities are a common finding in HLHS, even when adequately patched during the first stage of the Norwood procedure. This is thought to be due to the proliferation of residual ductal tissue. Residual systemic obstructive lesions might increase RV pressure, exacerbating valvular regurgitation, pulmonary vascular resistance, and resistance to venous drainage^21^.

While ventricular dysfunction was significantly higher in patients who developed PLE, ventricular function did not achieve statistical significance in multivariate regression, possibly due to the subjective methods by which ventricular function was assessed. Qualitative assessment of ventricular function might not be accurate in predicting mid- and long-term outcomes after Fontan. Zaidi and colleagues confirmed a similar finding in their study. This warrants the use of quantitative assessment, even using simple measures such as tricuspid annular plane systolic excursion, for grading of ventricular function in the univentricular heart. More advanced techniques, such as cardiac magnetic resonance (CMR), cannot be routinely used because of the need for sedation and cost-effectiveness^22^.

## Conclusions

It is important to share the peculiar experience of each center, as every surgical center has its own center and referral pool. The complexity of the cases referred to our center might explain the higher prevalence of complications. Some of the illustrated risk factors coincided with previous studies, notably a dominant systemic right ventricle and a larger conduit size. Others differed slightly, such as the cut-off for SVC pressures and the insignificance of the time lapse between Fontan and the development of complications. The lower cutoff point of SVC pressure might also be explained by the predominance of HLHS in the operated patients, with a higher likelihood of complications compared to other diagnoses.

We started a patient awareness campaign to draw attention to the families of our patients the absolute contraindications of completion of the Fontan pathway. We are trying to be more thorough in the patients undergoing Fontan and to reflect the findings of our study (especially AV valve regurgitation and ventricular dysfunction) into our patient selection.

More studies should be conducted to elucidate whether this cutoff for SVC pressure should replace the already settled cutoffs. In addition, further research is needed to develop new devices for the mechanical support of a failing Fontan.

## Acknowledgment

As the first author, I want to thank the residency program administrators in SKMC, notably Dr. Sareea Al Remeithi, for the outstanding residents’ program that has provided us with a great calibre of residents who were crucial to the authorship of this article.
